# Lipid–Metal-Organic Framework Colloidosomes for Multimodal Encapsulation and Pollutant Capture

**DOI:** 10.34133/csbj.0235

**Published:** 2026-10-14

**Authors:** Jeshua Podliska, Rahul Dev Jana, Raheleh Ravanfar

**Affiliations:** Department of Chemistry and Biochemistry, Texas Tech University, Lubbock, TX, USA.

## Abstract

The development of stable colloidosomes capable of accommodating chemically distinct molecular species while retaining accessible porosity remains an important challenge for multifunctional encapsulation and pollutant-capture applications. Here, we develop a hybrid lipid–metal-organic framework (lipid–MOF) colloidosome assembled through an interfacial emulsification strategy that integrates the structural rigidity of zeolitic imidazolate framework-8 particles with lipid-mediated membrane stabilization. During assembly, zeolitic imidazolate framework-8 particles accumulate at the oil–water interface to form a shell, producing hollow micron-sized spherical colloidosomes. The resulting colloidosomes exhibit excellent colloidal stability in aqueous media for more than 30 d with a zeta potential of approximately −50 mV. Nitrogen adsorption measurements reveal a surface area of 45 m^2^ g^−1^ and an average pore width of 4 nm for the dried composite following vacuum degassing at 100 °C for 24 h. Fluorescence imaging shows that hydrophobic Nile Red preferentially partitions into the colloidosomal membrane, whereas hydrophilic fluorescein isothiocyanate (t) localizes predominantly within the aqueous interior. The lipid–MOF colloidosomes enable encapsulation of hydrophilic methylene blue molecules with loading efficiencies approaching 90%. Furthermore, the colloidosomes demonstrate rapid removal of model pollutants from water, achieving >90% removal of methylene blue and metal ions without stirring. Together, these results introduce lipid–MOF colloidosomes as a new class of hybrid platforms that unify structural stability, multicompartmental encapsulation, and efficient adsorption behavior, opening pathways toward sustainable platforms for drug delivery application and environmental bioremediation.

## Highlights

• Hybrid lipid–MOF colloidosomes fabricated by interfacial assembly
• The colloidosomes remained stable in aqueous dispersion for more than 30 days.
• Multicompartment architecture enables dual-polarity molecular encapsulation
• Lipid reinforcement improves colloidosome stability in aqueous environments
• Hybrid colloidosomes remove >90% of dyes and metal ions without stirring
• Platform enables multifunctional bioprocessing and environmental applications

## Graphical Abstract

**Figure:**
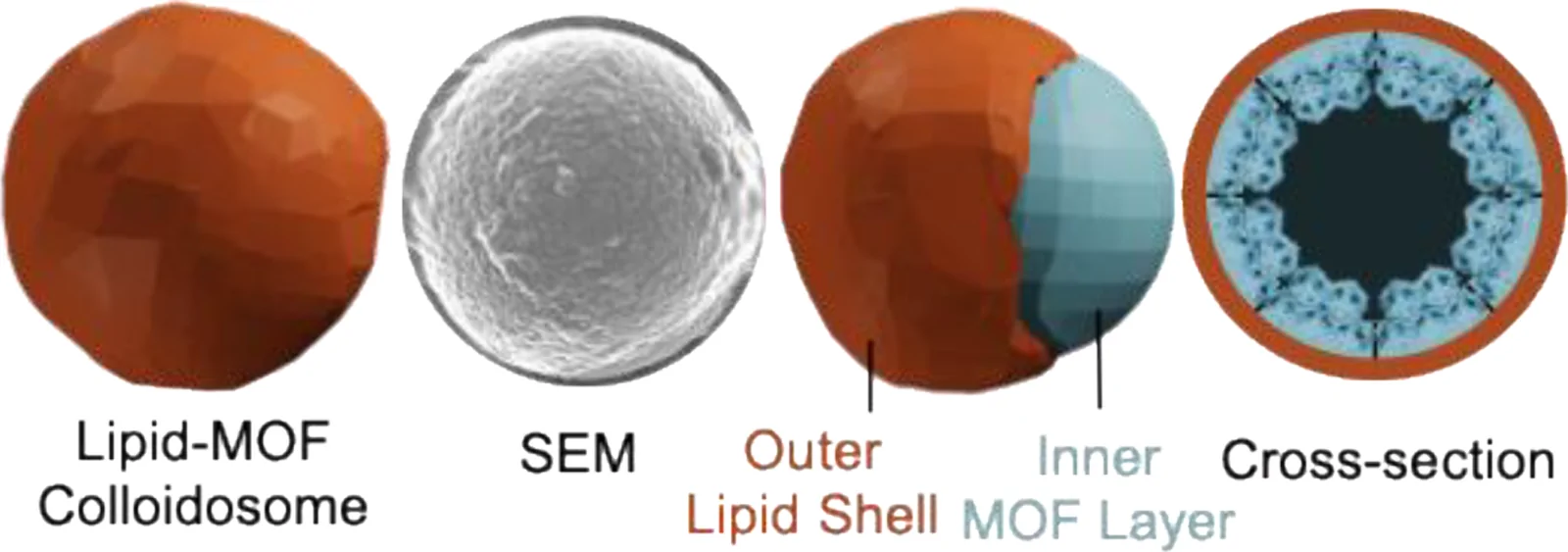

## Introduction

Colloidosomes, hollow microcapsules assembled from particles at emulsion interfaces, represent a versatile platform with tunable permeability, mechanical integrity, and chemical functionality [1–6]. Their hollow architecture enables the confinement of functional species within a protected interior while the surrounding shell regulates transport of molecules across the membrane. As a result, colloidosomes have been explored applications ranging from controlled delivery and catalysis to sensing and environmental remediation [2,7–11]. Despite these promising opportunities, achieving colloidosomes that simultaneously exhibit structural robustness, controlled permeability, and multifunctional encapsulation remains a persistent challenge. In many particle-based colloidosomes, the shell is formed through the jamming of nanoparticles at fluid interfaces, often producing interparticle defects or voids that can compromise mechanical stability and limit precise control over molecular transport [12]. Metal-organic frameworks (MOFs) have attracted substantial attention as building blocks for advanced colloidal assemblies because of their exceptional high surface areas, tunable pore structures, and chemical versatility [13,14]. Zeolitic imidazolate framework-8 (ZIF-8) has been widely studied owing to its chemical stability, accessible microporosity, and compatibility with aqueous room-temperature synthesis conditions [10,15–23]. Recent studies have demonstrated that MOF can assemble at liquid interfaces to generate MOF-based colloidosomes and hollow capsules with potential applications in catalysis, pollutant capture, and self-propelled micromotors [24–29]. While these approaches highlight the promise of MOF-derived colloidal shells, the resulting membranes typically consist of loosely packed nanocrystals, where interparticle gaps and structural heterogeneity may limit scalability and mechanical stability and reduce control over molecular partitioning within the capsule [2,30–35]. In biological systems, lipid membranes provide interfacial stability and polarity-dependent organization of molecules, while embedded proteins or inorganic cofactors introduce additional catalytic or transport functions. Inspired by these natural architectures, hybrid assemblies that combine lipid phases with inorganic materials can open new routes toward multifunctional compartmentalized systems.

Here, we engineered a cooperative lipid–MOF interfacial assembly strategy that enables the formation of lipid–MOF colloidosomes while preserving porous transport pathways across the shell. In this approach, ZIF-8 particles are incorporated during interfacial emulsification, while a thermally stable lipid phase co-assembles within the colloidosomal structure. Based on the assembly pathway, we propose that the lipid and ZIF-8 components form a hybrid interfacial shell; however, the precise nanoscale distribution and organization of these components within the shell are not directly resolved by the present characterization. Similar interfacial organization of ZIF-8 particles and formation of hollow ZIF-based structures have previously been reported in emulsion-derived and soft-templated systems [20,36]. Bian et al. [36] demonstrated interfacial growth of ZIF-8 nanoparticles at an oil/water Pickering-emulsion interface and obtained hollow ZIF-8-containing composites. Similarly, Singh et al. [20] demonstrated the spontaneous formation of hollow ZIF-8 colloidosomes through soft-templated assembly of ZIF-8 nanocrystals. Rather than forming a continuous impermeable coating, the solidifying wax domains bridge adjacent MOF particles, sealing structural defects and reinforcing shell cohesion while maintaining accessible porous transport throughout the colloidosomal mesoscopic voids. This cooperative organization produces a mechanically stabilized yet permeable hybrid interface. Using high-shear homogenization, this wax-assisted interfacial assembly process produces lipid–MOF colloidosomes with sustained colloidal stability in aqueous media. Because high-shear emulsification is compatible with larger-scale processing, the approach may potentially be amenable to scale-up, although scale-up was not evaluated in the present study. The resulting hierarchical architecture enables polarity-dependent spatial organization of guest molecules, where hydrophilic species localize preferentially within the aqueous interior and hydrophobic species partition into the lipid-integrated shell domain. Beyond molecular encapsulation, the aqueous interior functions as an accessible sequestration chamber for small organic molecules and metal ions. We investigated the ability of the colloidosome platform to remove methylene blue and selected metal ions as model species. Small molecule pollutants diffuse through the porous composite shell and accumulate within the internal cavity, where the confined geometry and large internal surface area enhance uptake efficiency. This core-mediated capture mechanism distinguishes our system from motion-driven or purely surface-adsorption MOF assemblies. Together, these results establish lipid–MOF colloidosomes as a new class of hybrid colloidal materials that integrate structural reinforcement, multicompartmental encapsulation, aqueous stability, and proof-of-concept pollutant capture, opening new avenues for potential applications in molecular delivery and environmental remediation.

## Results and Discussion

While MOFs have been extensively employed as stabilizers in Pickering emulsions [37,38], their cooperative assembly with a solid lipid phase to form structurally reinforced colloidosomes remains unexplored. We hypothesized that combining preformed MOF particles with a lipid matrix under double-emulsion conditions would enable the formation of hybrid colloidosomes, in which MOF particles and lipid components cooperatively organize at the emulsion interface (Fig. 1A). The schematic in Fig. 1A represents the proposed structural organization based on the sequential W/O/W assembly process rather than a directly resolved nanoscale architecture. To evaluate this concept, we apply a double-emulsion strategy in which an aqueous dispersion of preformed ZIF-8 was first emulsified within a molten lipid phase to generate a primary water-in-oil (W/O) emulsion, followed by secondary emulsification into an external aqueous stabilizer solution to produce water-in-oil-in-water (W/O/W) emulsions. Upon cooling, the lipid phase solidifies, yielding colloidosomal particles containing both lipid and MOF components within the hybrid interfacial structure. The precise nanoscale distribution of these components cannot be unambiguously distinguished by the present characterization and is therefore presented as a proposed structural model. Moreover, the stabilizers used in the secondary phase can play an important role in stabilizing these lipid–MOF colloidosomes. Because interfacial and structural stability of the colloidosomes is affected by both lipid composition and surfactant type, we screened various lipid–surfactant combinations to identify formulations capable of maintaining long-term colloidal stability (Table S1). Formulations prepared with olive oil and stabilized using hexadecyltrimethylammonium bromide (CTAB) (formulations #10 to 12) or guar gum (formulation #3) as stabilizers in the external aqueous phase exhibited visible phase separation within 1 to 2 d, indicating instability of the colloidosomal structures, insufficient interfacial stabilization, and subsequent coalescence under those particular compositions, rather than assigning a single molecular cause (Table S1). Stability in Pickering and mixed particle/surfactant emulsions depends strongly on particle wettability, interfacial coverage, surfactant identity and concentration, lipid viscosity, and resistance of the interfacial film to droplet coalescence. ZIF-8 itself has been shown to participate in stabilization of Pickering emulsions, with interfacial organization depending on the surrounding components [36]. Given the practical advantages of natural waxes, including renewable origin, biocompatibility with food and biomedical contexts, and thermal robustness during processing [39–45], we next focused on wax-based lipid matrices. Coconut wax provided improved structural integrity during emulsification and resisted coalescence during storage (Fig. 1B and Figs. S1 and S2). When combined with Triton X-100 (formulation #1) or Pluronic F127 (formulation #5) as the external stabilizer, the formulation yielded dispersions with a polydispersity index of 0.4 and 0.2, respectively, that remained colloidally stable for at least 38 d with no observable macroscopic phase separation (Fig. 1B and Figs. S1 and S2). In this formulation, ZIF-8 MOFs were first synthesized in aqueous solution by mixing 2-methylimidazole with zinc nitrate under stirring at room temperature, yielding a milky dispersion. This ZIF-8 aqueous dispersion was added dropwise to a molten lipid phase composed of coconut wax, soy lecithin, and glycerol monooleate maintained at 70 °C under stirring, forming a primary water-in-oil (W/O) emulsion. This primary emulsion was subsequently introduced dropwise into an external aqueous solution of stabilizer containing Triton X-100, Pluronic F127, or a combination of both under high-shear homogenization to generate a water-in-oil-in-water (W/O/W) double emulsion. Upon cooling to room temperature, the lipid phase solidified, producing colloidosomal particles in which ZIF-8 nanoparticles are organized along the solid lipid shell (Fig. 1A).

**Fig. 1.**
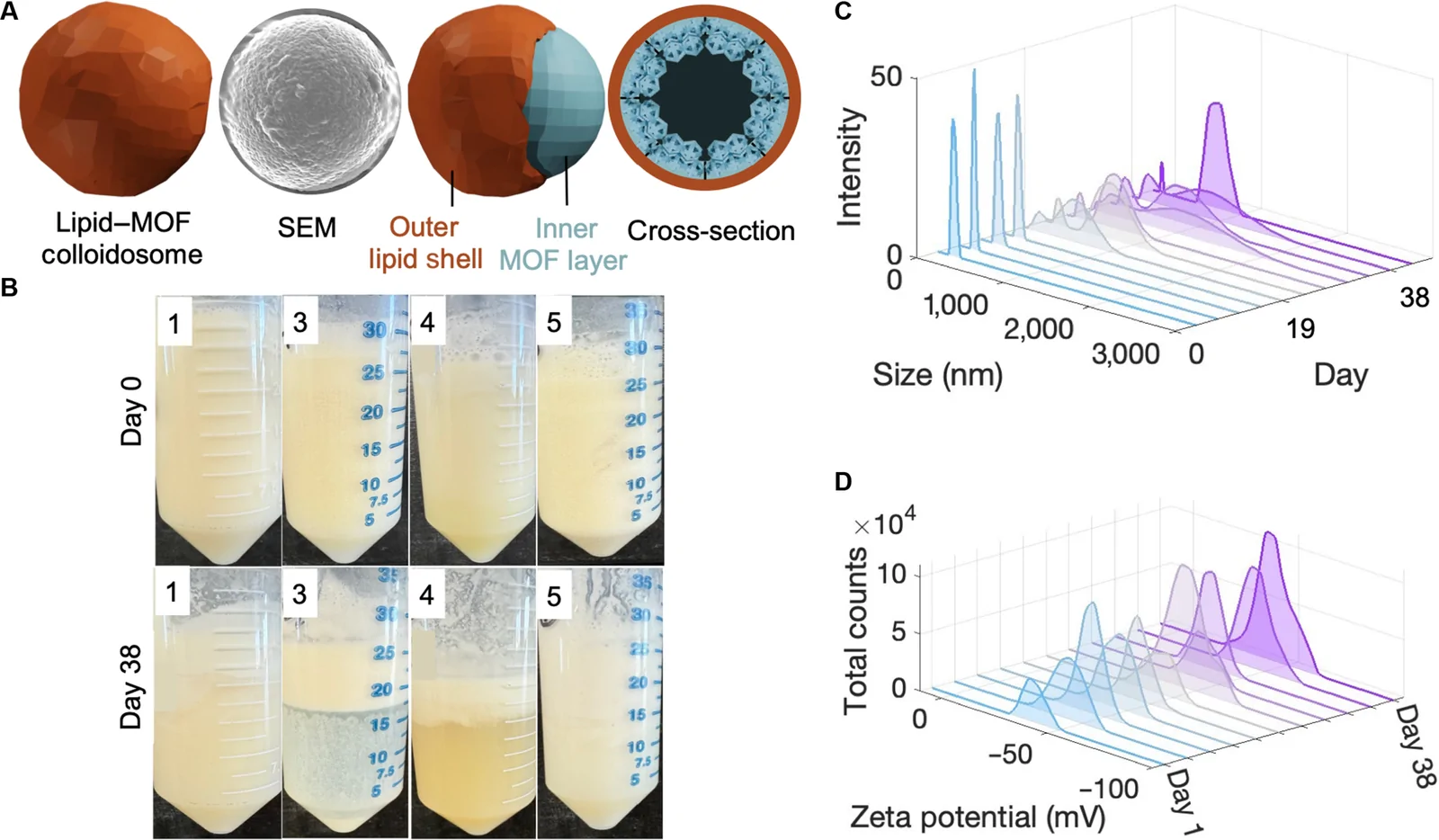
(A) Schematic representation of the proposed formation and structural organization of lipid–metal-organic framework (MOF) colloidosomes through the W/O/W interfacial emulsification process. SEM, scanning electron microscopy. The schematic illustrates the proposed organization of the lipid and zeolitic imidazolate framework-8 (ZIF-8) components and is not intended to represent a directly resolved nanoscale architecture. The inner aqueous phase, the lipid–MOF phase containing ZIF-8 and coconut wax/glycerol monooleate/soy lecithin, and the external aqueous phase containing stabilizer. (B) Representative images of different formulations, #1, #3, #4, and #5, showing their colloidal stability for more than 38 d (Table S1). Formulation #5, including Pluronic F127, showed stability for 38 d. (C) The hydrodynamic size for formulation #5, including Pluronic F127, for more than 38 d. (D) Zeta potential for formulation #5, including Pluronic F127, for more than 38 d.

The colloidal stability was evaluated by monitoring the hydrodynamic diameter and zeta potential over a 38-d storage period (Figs. S1 and S2). Dynamic-light-scattering analysis shows that the choice of stabilizer in the external aqueous phase significantly affects both the hydrodynamic diameter and polydispersity of the lipid–MOF colloidosomes (Fig. 1C and D and Figs. S1 and S2). The coconut wax/Triton X-100 formulations yielded particle sizes in the 2- to 4-μm range (Fig. S1). Replacing Triton X-100 with Pluronic F127 reduced the size of the lipid–MOF colloidosomes to 0.8 μm (Fig. 1C and Figs. S1 and S2), which confirms the exceptional role of Pluronic F127 in providing the hydrophilicity and steric stabilization compared to conventional nonionic surfactants [46,47]. The Pluronic F127-containing formulation exhibited a reported zeta potential of approximately −50 mV (Fig. 1D). Pluronic F127 is a nonionic poly(ethylene oxide)–poly(propylene oxide)–poly(ethylene oxide) triblock copolymer. Its expected stabilizing contribution is primarily steric, through an adsorbed polymer layer with hydrated chains extending into the aqueous phase [48]. The measured zeta potential reflects the electrokinetic behavior of the complete formulation under the measurement conditions. Its negative value cannot be assigned specifically to Pluronic F127. Ion adsorption and ionizable species associated with other formulation components are possible contributors. Our lipid–MOF colloidosomes demonstrated substantially enhanced stability compared to other reported ZIF-8 colloidosomes, which typically exhibit aggregation within 7 to 14 d [49]. Formulation #5 was selected as the most stable system and was further optimized with increasing the Pluronic F127 to 1% (formulation #6). This formulation was selected as the optimized formulation for physicochemical and structural characterization of the lipid–MOF colloidosomes. Application-specific variations of the lipid–MOF formulation were subsequently employed for the molecular encapsulation and pollutant-capture experiments, as detailed in the corresponding Methods sections. These formulations retained the same general lipid–MOF colloidosome design but differed in component ratios and/or surfactant concentration according to the experimental objective. Accordingly, formulation #6 should not be interpreted as the composition used for every subsequent experiment.

Fourier-transform infrared (FTIR) spectra and elemental analysis confirmed the successful integration of ZIF-8 in the lipid–MOF colloidosomes (Fig. 2B and Figs. S3 and S4). The spectrum of lipid–MOF colloidosomes shows the characteristic vibrational bands of ZIF-8, including the C=N stretching vibration of the imidazole ring at 1,584 cm^−1^ and Zn–N coordination vibration at 421 cm^−1^ [35], supporting the retention of characteristic ZIF-8-associated coordination and vibrational features following colloidosome formation (Fig. 2B). Energy-dispersive x-ray spectroscopy analysis further detected Zn in the lipid–MOF colloidosomes (Fig. S4), consistent with incorporation of the ZIF-8 component into the hybrid material. Because FTIR primarily probes local chemical bonding and functional-group vibrations, these observations do not independently establish preservation of the long-range crystalline order of ZIF-8. Accordingly, the FTIR results are interpreted as evidence for retention of characteristic ZIF-8-associated chemical features rather than as confirmation of framework crystallinity. In addition, the lipid–MOF spectrum illustrates bands at 2,920 cm^−1^ and 2,850 cm^−1^ corresponding to the asymmetric and symmetric stretching modes of aliphatic hydrocarbon chains from the wax, confirming the presence of the lipid phase within the hybrid structure (Fig. 2B). Thermogravimetric analysis was performed to evaluate the thermal profile of the lipid–MOF colloidosomes (Fig. 2A and Fig. S5). ZIF-8 shows a small initial mass loss below 200 °C, followed by gradual decomposition of the imidazolate linkers at higher temperatures while retaining a significant inorganic residue at 800 °C associated with zinc. In contrast, the lipid–MOF colloidosomes exhibit a substantial mass loss beginning at 250 °C and progressing rapidly between 300 and 420 °C, consistent with thermal degradation of the lipid component (Fig. 2A and Fig. S5). The significantly larger mass loss observed for lipid–MOF colloidosomes relative to ZIF-8 alone reflects both the dilution of the inorganic framework by the lipid phase and the modified decomposition pathway of lipid–MOF colloidosomes during thermal treatment. Scanning electron microscopy (SEM) images revealed the formation of spherical lipid–MOF colloidosomes with diameters in the 1- to 2-μm range (Fig. 2C to F). The smooth and continuous outer surface of the lipid–MOF colloidosomes indicates successful encapsulation of the MOF phase along with the lipid shell (Fig. 2C to F). Higher-magnification SEM images show partially fractured colloidosomes, which provide visual evidence of a hollow interior architecture formed through emulsion templating (Fig. 2E and F). In Fig. 2F, the observed surface morphology and contrast are consistent with formation of a heterogeneous lipid–MOF composite structure. However, SEM does not provide sufficient nanoscale resolution or compositional contrast to unambiguously assign discrete lipid-rich and MOF-rich layers. This morphology differs significantly from conventional MOF colloidosomes assembled solely from aggregated crystalline MOFs, which typically exhibit rough, granular shells with visible interparticle voids [19,50]. Instead, the lipid–MOF colloidosomes display a smoother and more cohesive shell structure (Fig. 2C to F). This behavior is attributed to lipid matrix which fills interstitial spaces between ZIF-8 particles and produces a more continuous composite shell.

**Fig. 2.**
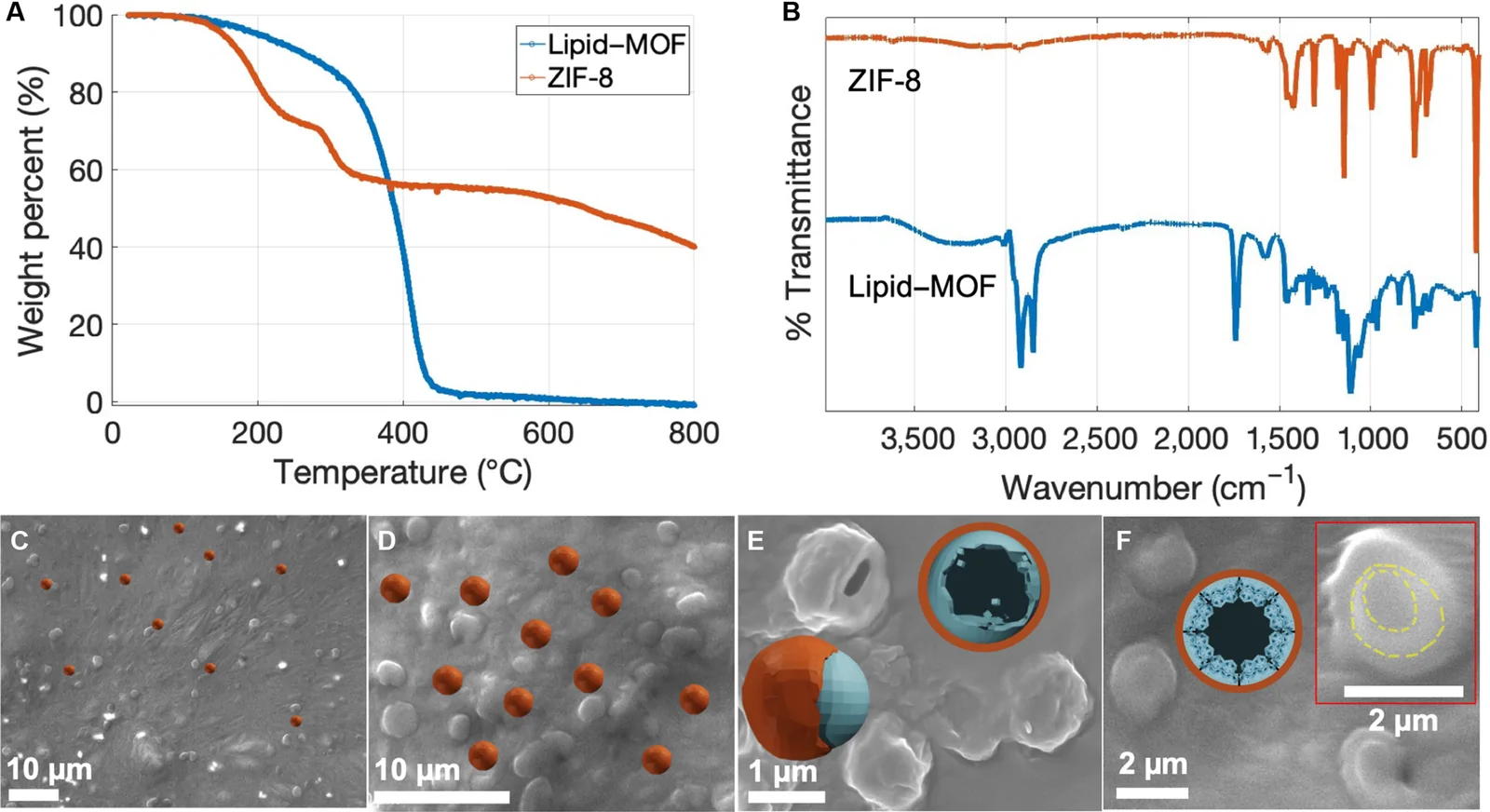
(A) Thermogravimetric analysis for the lipid–MOF colloidosomes and ZIF-8. (B) Fourier-transform infrared spectra for lipid–MOF colloidosomes and ZIF-8. (C) SEM image of the lipid–MOF colloidosomes that appeared well dispersed. (D) Higher-magnification SEM image of the lipid–MOF particles. (E) The fractured lipid–MOF colloidosome with a hollow interior domain. (F) Contrast variations within the lipid–MOF colloidosomes, illustrating the surface lipid–MOF layer, and hollow inner domain.

An important feature of the lipid–MOF colloidosomes is their ability to spatially compartmentalize molecules according to their polarity, enabling the coexistence of hydrophobic and hydrophilic species within distinct domains of the same carrier. To evaluate this compartmentalization behavior, we investigated the encapsulation of fluorescent probes with contrasting polarity using fluorescein isothiocyanate (FITC) as a hydrophilic model compound and Nile Red as a hydrophobic probe. Confocal laser scanning microscopy revealed different fluorescence patterns in the 2 imaging channels (Fig. 3A). Nile Red exhibited strong red fluorescence predominantly at the lipid–MOF colloidosomal shell due to its lipophilic nature (Fig. 3A). In contrast, FITC displayed yellow fluorescence primarily within the interior of the particles, indicating accumulation in the aqueous cavity of the colloidosomes (Fig. 3A). Overlay images further show peripheral red fluorescence surrounding an interior yellow signal. The distinct fluorescence distributions are consistent with preferential localization of Nile Red toward the lipid-rich interfacial region and FITC toward the aqueous interior, rather than demonstrating complete phase-exclusive partitioning (Fig. 3A). Importantly, preferential localization of hydrophilic and hydrophobic fluorophores in distinct regions of multiphase microcapsules has strong literature precedent. Kim et al. [51] reported W/O-derived multifunctional microcapsules in which FITC-labeled dextran incorporated into the aqueous phase remained primarily in the aqueous core, whereas Nile Red introduced through the hydrophobic phase localized predominantly in the shell region. Such spatial segregation within a single microcarrier motivates the potential of lipid–MOF colloidosomes as versatile platforms for co-encapsulation, controlled release, and multifunctional systems [52–59].

**Fig. 3.**
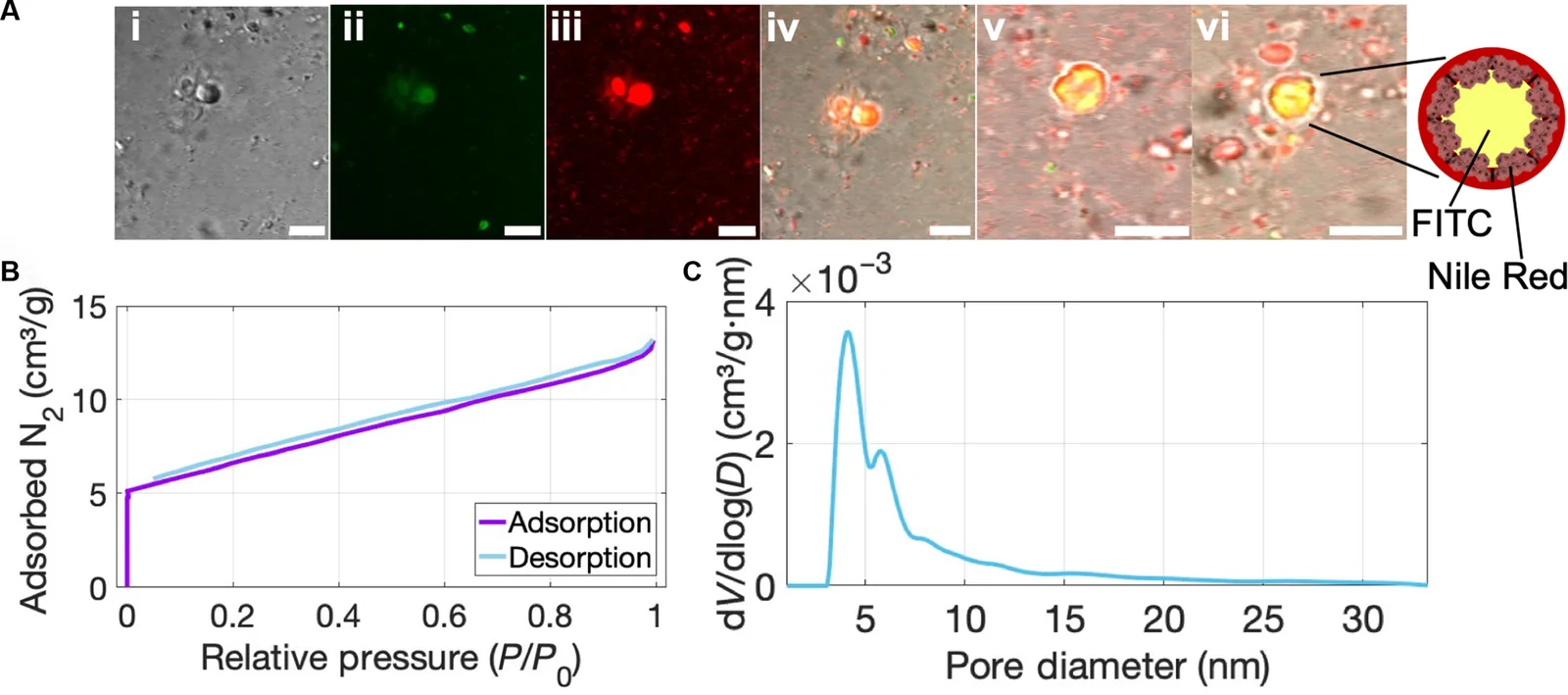
(A) Confocal laser scanning microscopy images of lipid–MOF colloidosomes. i: Bright field; ii: excited at 488 nm; iii: excited at 561 nm; iv, v, and vi: overlay images show the lipid–MOF colloidosomes with red shell and yellow core due to the accumulation of Nile Red and fluorescein isothiocyanate (FITC), respectively. Scale bars: 10 μm. (B) Nitrogen adsorption and desorption curves. (C) Density functional theory pore-size distribution detected with N_2_ adsorption and desorption at 77 K for lipid–MOF colloidosomes.

Nitrogen adsorption measurements revealed a Brunauer–Emmett–Teller (BET) surface area of 45 m^2^ g^−1^ and a pore volume of 0.02 cm^3^ g^−1^ for the lipid–MOF colloidosomes following vacuum degassing at 100 °C for 24 h (Fig. 3B, Fig. S6, and Table S2), substantially lower than values typically reported for pristine ZIF-8. This reduction may arise from incorporation of the lipid phase, partial obstruction of accessible ZIF-8 surfaces, altered interparticle packing, and structural changes associated with drying and activation, all of which may reduce nitrogen accessibility under cryogenic adsorption conditions. Contributions may also include mass dilution by the lipid and other formulation components, partial blocking of nitrogen-accessible surfaces, altered interparticle packing, and structural reorganization during drying and degassing. Their individual contributions were not resolved by the present measurements. The average pore diameter obtained from density functional theory (DFT) analysis (~4 nm) suggests that the accessible porosity is dominated by mesoporous domains arising from interparticle spaces rather than the intrinsic microporosity of ZIF-8 (Fig. 3C and Table S2).

Wastewater generated from industrial processes rarely contains a single type of contaminant; instead, it typically consists of complex mixtures of organic dyes, dissolved metal ions, and other chemical species. Despite this reality, most remediation studies still rely on simplified model systems that examine the removal of only one pollutant class at a time. The release of synthetic dyes into natural water systems is a particularly pressing environmental concern because these compounds are often chemically stable, highly visible even at low concentrations, and potentially harmful to both ecosystems and human health [60,61]. Methylene blue (MB), for example, is widely used in industrial dyeing and biological staining processes, yet its accumulation in water sources has been associated with adverse health effects including respiratory irritation, gastrointestinal distress, and eye irritation [62–64]. Materials capable of addressing multiple contaminant types simultaneously would therefore represent a meaningful step toward more practical wastewater treatment technologies. In the present work, we explored whether lipid–MOF colloidosomes could serve as versatile adsorption platforms capable of removing both organic dyes and metal ions from aqueous environments. Given the hierarchical structure and amphiphilic interfaces present in our lipid–MOF colloidosomes, we reasoned that these assemblies might provide an efficient platform for dye capture. Indeed, when exposed to aqueous MB solutions, the colloidosomes demonstrated near-complete removal of the dye without stirring (Fig. 4A). Dynamic-light-scattering measurements revealed a clear increase in particle size after dye exposure, reaching approximately 4 μm, which suggests that MB molecules either adhere to the outer surface or are taken up into the hollow interior of the assemblies (Fig. S7). Moreover, the adsorption without stirring was more efficient than with stirring (Fig. S8). The comparison demonstrates a dependence on the experimental mixing conditions but does not identify the underlying capture mechanism. Surface association and partitioning into formulation components remain possible contributions that were not distinguished by these measurements.

**Fig. 4.**
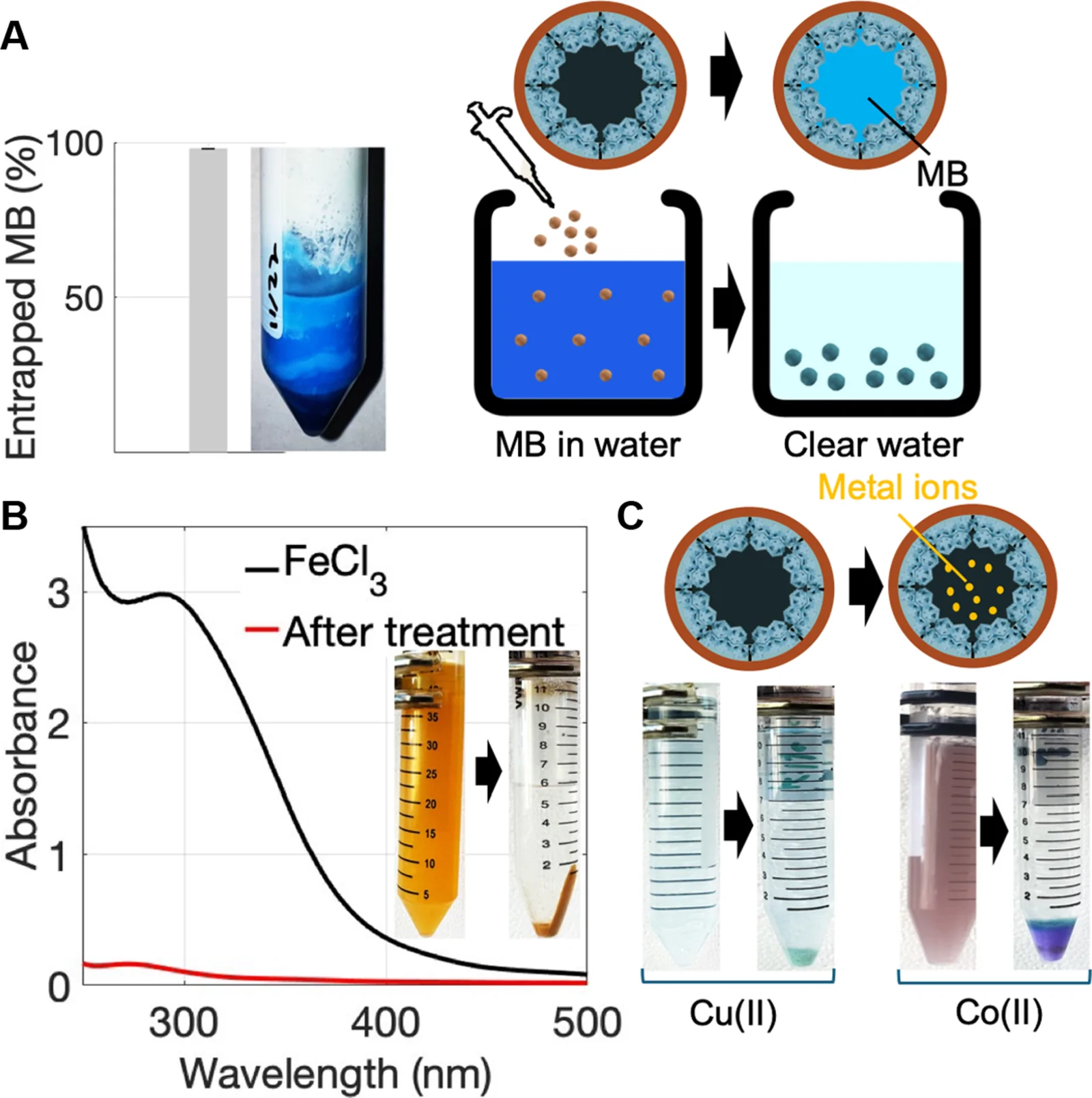
(A) Apparent encapsulation efficiency for methylene blue (MB) introduced during colloidosome formation and apparent MB removal by preformed colloidosomes, determined in separate experiments. (B) UV-vis spectra and optical images illustrating the Fe(III) removal capacity of lipid–MOF colloidosomes without stirring. (C) Optical images of Cu(II) and Co(II) removal capacity of lipid–MOF colloidosomes.

In a separate experiment, MB was introduced into the primary aqueous phase during colloidosome preparation. Apparent encapsulation efficiency was estimated indirectly by measuring residual MB in the supernatant after centrifugation (Fig. 4A). This experiment evaluates incorporation during synthesis and is distinct from removal by preformed colloidosomes. The encapsulation efficiency of MB in the lipid–MOF colloidosomes was 90 ± 6%, which shows the intriguing capability of these colloidosomes for entrapping the hydrophilic molecules (Fig. 4A). The indirect measurement does not establish whether retained MB resides within the cavity or at the particle surface.

Heavy metal contamination represents another major environmental challenge associated with industrial activity. Metals such as mercury, cadmium, lead, chromium, and arsenic can persist in aquatic systems and pose serious risks due to their toxicity and ability to bioaccumulate [65,66]. To explore whether the lipid–MOF colloidosomes could also address this class of pollutants, we investigated their ability to capture dissolved metal ions using Fe(III), Cu(II), and Co(II) as representative examples. Upon introduction of the colloidosomes into a solution containing FeCl_3_ (1,000 ppm), CoSO_4_ (100 ppm), or CuCl_2_ (100 ppm), the lipid–MOF colloidosomes rapidly interacted with the metal-containing environment without stirring, accompanied by a visible color change that indicated metal uptake (Fig. 4B and C and Videos S1 to S3). The lipid–MOF colloidosomes gradually aggregated and settled to the bottom of the solution after the adsorption process was complete (Fig. 4B and C and Videos S1 to S3). Quantitative ultraviolet–visible (UV-vis) analysis confirmed that more than 90% of the iron, cobalt, and copper were removed from solution (Fig. 4B and C and Figs. S9 and S10), suggesting that the reduced BET surface area does not preclude efficient adsorption in aqueous environments. Instead, the hybrid architecture of lipid–MOF colloidosomes introduces interfacial adsorption sites and mesostructured pathways that facilitate the capture of small organic molecules and metal ions. This platform may provide a promising starting point for the development of colloidal assemblies capable of capturing a broader range of heavy metal contaminants and organic dyes from wastewater [67].

The present results establish an initial basis for investigating lipid–MOF colloidosomes, but their suitability for biomedical applications remains untested. The absence of visible phase separation during storage does not establish physiological stability or biological compatibility. Previous studies have demonstrated medium-dependent changes in ZIF-8 structure and cargo retention in laboratory buffers, cell-culture media, and serum [68]. These findings motivate direct evaluation of the present composite; they do not establish its degradation behavior or demonstrate that lipid incorporation protects the framework. Biomedical assessment would require measurements of framework stability, zinc and linker release, cytotoxicity, and interactions with proteins and cells under conditions relevant to the intended application. Furthermore, probe incorporation and fluorescence localization do not establish controlled release, cargo bioactivity, or therapeutic delivery performance. For water-treatment applications, the experiments were conducted with individual model contaminants and do not establish selectivity or performance in mixed-contaminant solutions or real wastewater. Competing solutes, dissolved organic matter, pH, and ionic strength should be evaluated because performance under these conditions cannot be inferred from the present tests. Practical assessment would also require validated uptake capacities and recovery of the colloidosomes from treated water. Regeneration and reuse were not evaluated in the present proof-of-concept study. The development of suitable regeneration procedures and assessment of performance over repeated treatment cycles therefore remain important future directions. Potential release of zinc, the organic linker, surfactants, and other formulation components must also be measured to determine whether treatment introduces additional substances into the water. The present work therefore provides a proof-of-concept materials platform whose application-specific performance and safety remain to be established.

## Conclusion

In this work, we developed hybrid lipid–MOF colloidosomes through an interfacial emulsification strategy that brings together the structural robustness of ZIF-8 with the stabilizing properties of a solid lipid phase. This cooperative assembly produces hollow colloidosomes with diameters of approximately 1 to 2 μm that remain colloidally stable in aqueous environments for extended periods. The architecture of these colloidosomes enables spatial organization of molecules according to their polarity. Confocal fluorescence imaging shows that hydrophobic Nile Red preferentially associates with the lipid-rich shell, while hydrophilic FITC accumulates within the aqueous interior. This behavior highlights the ability of the hybrid structure to accommodate molecules with different chemical characteristics within separate domains of the same particle. In addition, MB incorporated during colloidosome formation exhibited an encapsulation efficiency approaching 90%. Nitrogen adsorption measurements further suggest that the accessible porosity of the system is largely associated with mesoscopic interparticle spaces within the colloidosomal shell. Beyond encapsulation, the lipid–MOF colloidosomes also exhibit promising adsorption behavior toward removal of model aqueous contaminants. The particles efficiently removed more than 90% of methylene blue dye and individually tested representative metal ions such as Fe(III), Cu(II), and Co(II) from solution without the need for stirring. These observations demonstrate the ability of the lipid–MOF formulations to capture chemically distinct dissolved species under the tested conditions, although the relative contributions of the MOF, lipid, and interfacial components and the precise capture mechanisms remain to be established. Overall, these results demonstrate that combining lipid phases with MOF particles at emulsion interfaces offers a simple route to multifunctional colloidal assemblies. The resulting lipid–MOF colloidosomes integrate colloidal stability, compartmentalized encapsulation, and proof-of-concept pollutant capture within a single platform. Further studies of cargo release, selectivity, regeneration, and performance in complex aqueous media will be required to establish application-specific performance. With these considerations, lipid–MOF colloidosomes provide a useful foundation for future development of molecular delivery and environmental remediation technologies. Related lipid/ZIF-8 architectures illustrate additional directions for investigation. Cano-Sarabia et al. [35] reported liposome- and vesicle-based ZIF-8 biocomposites for hydrophobic-cargo delivery, including evaluation of astaxanthin release. Qin et al. [69] developed vitamin-A-modified ZIF-8/lipid nanoparticles for pirfenidone delivery in liver-fibrosis therapy. Although these formulations differ structurally from the present colloidosomes, they motivate investigation of lipid organization and surface functionalization as design variables for future delivery systems. Incorporation of suitable catalytic or molecular-recognition components could also be explored for catalysis and sensing.

## Materials and Methods

### Materials

Pluronic F127, 2-methylimidazole, Span 80, CTAB, copper (II) chloride, iron (III) chloride, and cobalt (II) sulfate heptahydrate were obtained from Sigma-Aldrich, MO, USA. Triton X-100, fluorescein isothiocyanate, Nile Red, MB, and palmitic acid were obtained from Thermo Fisher Scientific, MA, USA. Zinc nitrate hexahydrate and nickel sulfate were obtained from Ward’s Science, ON, Canada. Coconut wax was obtained from American Soy Organics, IA, USA. Liquid soy lecithin was obtained from Velona, IL, USA. Glycerol monooleate was obtained from Spectrum, NJ, USA. Pure olive oil was obtained from Albertsons Companies, ID, USA.

### Preparation of lipid–MOF colloidosomes

Briefly, 1 mL of 0.1 M zinc nitrate was added to 1 mL of 2.5 M 2-methylimidazole while stirring at 1,000 rpm for 20 min at room temperature to form a milky white dispersion of ZIF-8 MOF. The resulting ZIF-8-containing aqueous dispersion was used directly for the subsequent emulsification step without isolation or drying. Therefore, an isolated ZIF-8 yield was not determined for this preparation. The lipid phase, containing coconut wax (1.2 g), soy lecithin (0.35 g), and glycerol monooleate (GMO) (0.35 g), was heated to 70 °C and stirred at 1,000 rpm for 10 min. The preformed MOF solution was then added dropwise to the lipid phase and left to stir for 10 min forming the W/O emulsion. The resulting emulsion was diluted to an aqueous phase consisting of Triton X-100 (1% w/w) or Pluronic F127 (0.5% w/w) under high-shear homogenization at 15,000 rpm for 15 min to generate W/O/W double emulsion while the lipid is solidified at room temperature to form the lipid shell, where the ZIF-8 particles are oriented along with the lipid shell at the emulsion interface. In the sequential W/O/W process, soy lecithin and GMO are incorporated into the lipid phase and participate primarily in stabilization and organization of the internal water/lipid interface, whereas Pluronic F127, Triton X-100, CTAB, or guar gum, depending on the formulation, are introduced through the external aqueous phase and contribute primarily to stabilization of the outer lipid/water interface. The resulting colloidosomes were dried using speedvac (Thermo Fisher Savant Speed Vac Plus SC110A; Savant Instruments Inc., USA) before subsequent characterization.

### Confocal laser scanning microscopy

To investigate partitioning within the lipid–MOF colloidosomes, Nile Red and FITC were used as fluorescent probes for hydrophobic and hydrophilic domains, respectively. Nile Red preferentially stains the lipid-rich shell, whereas FITC localizes within the internal aqueous cavity, enabling visualization of the spatial organization of hydrophobic and hydrophilic regions within the colloidosomal architecture. Confocal imaging was performed using an Olympus FV3000 confocal laser scanning microscope (Olympus Corp., Tokyo, Japan) equipped with 100× objective lenses. We added 40 μL of 0.1% (w/w) FITC and 0.1% (w/w) Nile Red per 1-mL emulsion and stirred for 30 min in the dark [70]. Fluorescence images were acquired using FITC (*λ*_ex_: 488 nm) and propidium iodide excitation (*λ*_ex_: 561 nm) and emission filter sets, along with differential-interference-contrast imaging.

### Dynamic light scattering

Particle-size distribution of lipid–MOF colloidosomes was monitored using Malvern Zetasizer Pro (Malvern Panalytical Ltd, UK) in different formulations. The colloidal stability of the particles was assessed through zeta-potential measurements with an electroactive cell (DS1070). Measurements were performed at 25 °C and at the native sample medium. Samples were equilibrated in the instrument for 30 s before measurement. For each sample, 3 consecutive instrumental measurements were acquired and used to determine the mean hydrodynamic diameter and polydispersity index. These measurements represented repeated readings of the same preparation.

### Thermogravimetric analysis

Thermal stability of lipid–MOF colloidosomes was assessed using TGA (Shimadzu DTG-60H; Shimadzu Corp., Kyoto, Japan). Emulsions were centrifuged at 20,000×*g* for 30 min and the pellet was dried using SpeedVac. The dried samples were placed in alumina crucibles (AL6211 6 mm) and heated from room temperature to 800 °C under a nitrogen atmosphere at a heating rate of 10 °C min^−1^.

### Attenuated total reflectance–FTIR spectroscopy

Chemical composition of the emulsions was analyzed using attenuated total reflectance–FTIR spectroscopy (Nicolett FTIR, ThermoFisher Scientific, Thermo Electron Scientific Instruments LLC, USA).

### UV-vis spectrophotometry

UV-vis spectrophotometry was conducted in the range of 190 to 900 nm, with 1.0-nm data interval and 0.004-s averaging time, on an Agilent Cary UV-Vis Compact Peltier from Agilent Technologies, Mulgrave, Australia.

### SEM and elemental analysis

Surface morphology was investigated by SEM using a Zeiss crossbeam 540 focused ion beam–SEM equipped with an Oxford energy-dispersive x-ray spectroscopy system from ZEISS, 07745 Jena, Germany, for elemental analysis. The SEM images were collected at 5-kV accelerating voltage and 5.9-mm working distance with side-mount secondary electron detector. Samples were freeze dried using a Labconco Freezone (Labconco Corp., Kansas City, MO, USA) for 48 h, placed on aluminum stubs covered with carbon adhesive tape, and sputter-coated with iridium using a Technics Hummer V Sputter Coater (Anatech USA, Union City, CA, USA).

### BET analysis

Surface area analysis was performed using N_2_ adsorption–desorption isotherms at 77 K with a Quantachrome ASiQwin analyzer (Quantachrome Instruments, USA). Prior to measurements, samples were degassed under vacuum at 100 °C for 24 h to eliminate adsorbed gases and moisture. The measurements therefore characterize the dried and degassed composite. Preservation of the original hydrated colloidosome structure during this treatment was not established, and redistribution of the lipid phase or other structural changes cannot be excluded. The specific surface area of the samples was determined using the BET method. The BET equation was applied in the relative pressure range (*P*/*P*_0_) of 0.05 to 0.50, where the isotherms showed linearity. The surface area was calculated based on the measured adsorption data. Pore-size distribution and pore volume were further analyzed using DFT applied to the N_2_ adsorption–desorption isotherms. The analysis was carried out using N_2_ at 77 K on carbon (cylindrical pores, QSDFT adsorption branch). Model selection was based on the expected pore shape determined from SEM. Robustness to kernel selection and compliance of the selected BET interval with consistency criteria were not established.

### Encapsulation efficiency of small molecules in lipid–MOF colloidosomes

In order to investigate the capability of lipid–MOF colloidosomes for encapsulating the hydrophilic small molecules, MB was used as a model compound during colloidosome formation. Briefly, 0.05 g MB was dissolved in the primary aqueous phase containing the precursors of ZIF-8 and applied during the formation of double emulsions as explained in the previous section. The lipid–MOF colloidosomes were then centrifuged and the MB was measured at 664 nm using MB standard curve in the supernatant using Cary 60 UV-vis spectrophotometry (Agilent Technologies, USA). An indirect estimate of apparent encapsulation efficiency was obtained from the difference between the initial MB mass and the MB mass measured in the supernatant. Experiments were performed using 3 independently prepared batches. Repeated measurements within each batch were averaged before calculating the overall mean and standard deviation across independent batches.

$$EE_{\mathrm{MB}}\;\left(\%\right)=\frac{{\mathrm{MB}}_{\text{added}}-{\mathrm{MB}}_{\text{free},\text{measured}}}{{\mathrm{MB}}_{\text{added}}}\times 100$$ (1)

### Removal of small molecules using lipid–MOF colloidosomes

MB removal by preformed colloidosomes was tested in this experiment. The best removal efficiency for MB was associated to the sample that was synthesized using 10 mL 0.1 M zinc nitrate, 10 mL of 2.5 M 2-methylimidazole, 3% (w/w) Pluronic F127, 0.6 g coconut wax, and 0.15 g soy lecithin and GMO. Briefly, 1 mL of preformed colloidosomes was added to 2-, 4-, and 8-mL solution of 500 ppm MB for 1 h, with or without stirring. The samples were then centrifuged at 20,000×*g* and the supernatant was analyzed by UV-vis spectrophotometry to monitor the residual of MB and used to calculate the removal capacity of lipid–MOF colloidosomes with and without stirring. This experiment evaluated apparent MB removal from the aqueous phase, rather than incorporation during colloidosome synthesis. Removal percentage alone does not identify the location of retained dye. Experiments were performed using 3 independently prepared batches. Repeated measurements within each batch were averaged before calculating the overall mean and standard deviation across independent batches.

$${\text{Removal}}_{\mathrm{MB}}\;\left(\%\right)=\frac{m_{\mathrm{MB},\text{initial}}-m_{\mathrm{MB},\text{aqueous}}}{m_{\mathrm{MB},\text{initial}}}\times 100$$ (2)

### Removal of metal ions using lipid–MOF colloidosomes

The best removal efficiency for metal ions was associated to the sample that was synthesized by preparing the lipid phase mixed with hexane and Span 80 and using 3 mL of 0.1 M zinc nitrate and 3 mL 2.4 M 2-methylimidazole. Briefly, 1 mL of this sample was added to 6-mL aqueous solution of iron(III) chloride (1,000 ppm), or copper(II) chloride (100 ppm), or cobalt(II) sulfate (100 ppm) and incubated for 1 h without stirring. Then, the samples were centrifuged and the supernatant was monitored for the trace of metal ions using UV-vis spectrophotometry to assess changes in the absorption spectra following treatment. The reported concentrations refer to the starting metal–salt solutions before addition of the colloidosome dispersion. Because different starting concentrations were used, the experiments are considered separately and do not establish comparative metal-ion selectivity. Experiments were performed using 3 independently prepared batches. Repeated measurements within each batch were averaged before calculating the overall mean and standard deviation across independent batches.

## Data Availability

The supplementary information, raw data, and custom analysis scripts are available upon request from the corresponding author.
