# Supplementary material for: Lipid–Metal-Organic Framework Colloidosomes for Multimodal Encapsulation and Pollutant Capture
Source: Comput Struct Biotechnol J. 2026 Oct 14;35(3):0235. doi: 10.34133/csbj.0235 (PMC13662271; doi:10.34133/csbj.0235)
Supplement: Supplementary file 1 [file csbj.0235.f1.zip › SI-Revised.docx]

**Lipid–MOF Colloidosomes for Multimodal Encapsulation and Pollutant Capture**

Jeshua Podliska, Rahul Dev Jana, Raheleh Ravanfar*

Department of Chemistry and Biochemistry, Texas Tech University

Table S1. Formulations evaluated during screening for colloidal stability.

| **Formulation** | **Oil phase** | **Surfactant in Oil Phase** | **Surfactant in Aqueous Phase** |
| --- | --- | --- | --- |
| 1 | Coconut Wax | GMO + Soy Lecithin | Triton X-100 (0.5%) |
| 2 | Coconut Wax | GMO + Soy Lecithin | Triton X-100 (1%) |
| 3 | Coconut Wax | GMO + Soy Lecithin | Triton X-100 + Guar Gum |
| 4 | Bees Wax | GMO + Soy Lecithin | Triton X-100 |
| 5 | Coconut Wax | GMO + Soy Lecithin | Pluronic acid (0.5%) |
| 6 | Coconut Wax | GMO + Soy Lecithin | Pluronic acid (1%) |
| 7 | Palmitic Acid | GMO + Soy Lecithin | Triton X-100 |
| 8 | Coconut Wax | GMO + Soy Lecithin | Triton X-100 + Alginate |
| 9 | Palmitic Acid | GMO + Soy Lecithin | Triton X-100 + Alginate |
| 10 | Olive Oil | GMO | CTAB |
| 11 | Olive Oil | GMO + Soy Lecithin | CTAB |
| 12 | Olive Oil | Soy Lecithin | CTAB |
| 13 | Hexane | Span® 80 | Pluronic acid |

Table S2. Surface area, pore width, and pore volume of lipid-MOF colloidosomes derived from multipoint Brunauer–Emmett–Teller (BET) and Density Functional Theory (DFT) analysis.

| Pore Volume | 0.018 cc/g |
| --- | --- |
| Surface area | 45.9 m^2^/g |
| Lower confidence limit | 1.066 nm |
| Fitting Error | 1.503 % |
| Average Pore Width | 4.1 nm |

Figure S1. Particle size measurements over 38 days for formulations a) #5, and b) # 2.

Figure S2. Zeta Potential measurements over 38 days for formulations a) # 1, b) # 5.

Figure S3. FTIR spectra for lipid-MOF colloidosomes synthesized through formulations #1, #5, and #6, ZIF-8, and lipid nanoparticles (LNP control).

Figure S4. Elemental analysis confirms the presence of zinc in the lipid-MOF colloidosomes.

Figure S5. TGA for the lipid-MOF colloidosomes synthesized through formulations #4, #5, #6, #7, ZIF-8, and lipid nanoparticles (LNP control).

Figure S6. Multi-point BET plot of pore size distribution, showing that Pluronic F127 carries a distinct mesoporous structure.

Figure S7. Increasing the size of lipid-MOF samples after adsorption of MB.

Figure S8. Removal of MB from 2, 4, and 8 mL solution of 500 ppm methylene blue using 1 mL of lipid-MOF colloidosomes after 1 hour with stirring (gray color, flowed) and without stirring (blue color, passive).

Figure S9. UV-vis spectra illustrating the Cu(II) adsorption capacity of lipid-MOF colloidosomes without stirring.

Figure S10. UV-vis spectra illustrating the Co(II) adsorption capacity of lipid-MOF colloidosomes without stirring.

**Supplementary Video Legends**

Movie S1. Time-lapse video showing the rapid removal of Fe^3+^ ions from aqueous solution by lipid–MOF colloidosomes through adsorption.

Movie S2. Time-lapse video showing the rapid removal of Cu^2+^ ions from aqueous solution by lipid–MOF colloidosomes through adsorption.

Movie S3. Time-lapse video showing the rapid removal of Co^2+^ ions from aqueous solution by lipid–MOF colloidosomes through adsorption.
